# Depth‐dependent hydration dynamics in human skin: Vehicle‐controlled efficacy assessment of a functional 10% urea plus NMF moisturizer by near‐infrared confocal spectroscopic imaging (KOSIM IR) and capacitance method complemented by volunteer perception

**DOI:** 10.1111/srt.13137

**Published:** 2022-01-15

**Authors:** Julia Gallinger, Andreas Kuhn, Sonja Wessel, Peter Behm, Silke Heinecke, Alexander Filbry, Linus Hillemann, Frank Rippke

**Affiliations:** ^1^ Research and Development Beiersdorf AG Hamburg Germany

**Keywords:** corneometry, dry skin, glycerol, hydration gradient, natural moisturizing factor, perceived moisturization, stratum corneum, xerosis

## Abstract

**Background:**

Stratum corneum (SC) hydration is vital for the optimal maintenance and appearance of healthy skin. In this context, we evaluated the efficacy of an NMF‐enriched moisturizer containing 10% urea on different aspects of SC hydration of dry skin.

**Material and Methods:**

In two clinical studies, the hydration efficacy of the moisturizer in comparison to its vehicle was investigated. In the first study, 42 subjects applied the moisturizer and the vehicle to one lower leg each. Thirty minutes and 24 h after this single treatment, SC hydration was measured by corneometry. Volunteers also rated skin moisturization and evaluated product properties. In the second study, 27 subjects each treated one forearm twice daily for 2 weeks with the moisturizer and the vehicle. Then, depth‐resolved water‐absorption spectra were measured by near‐infrared confocal spectroscopic imaging (KOSIM IR).

**Results:**

The moisturizer exerted a superior hydrating effect compared to the vehicle. KOSIM IR measurements show that, compared to the vehicle, the moisturizer significantly improved the water gradient in the SC from the surface to a depth of 15 μm. Moreover, the moisturizer received high acceptance ratings from the volunteers and was preferred to the vehicle.

**Conclusion:**

The humectants applied in the investigated moisturizer improved SC water content in total and as a function of depth. The combination of depth‐resolved data (KOSIM IR) with classical corneometry provides an integrated concept in the measurement of skin hydration, rendering both methods complementary. These findings were in line with the volunteers` self‐assessments of the moisturizer properties that are relevant to treatment adherence.

## 1 INTRODUCTION

The stratum corneum (SC) represents the outermost layer of the epidermis establishing a protective barrier against the external environment. For most body areas, SC thickness is reported to be around 20 μm.^1^, ^2^, ^3^ Although it constitutes a nonviable tissue, the SC plays an essential role in regulating skin moisturization and water retention. Its water content decreases from a constant level of about 70% in the stratum granulosum to about 15–25% at the skin surface.^4^, ^5^, ^6^, ^7^, ^8^ Hydration of the SC is a key factor for desquamation, generation of natural moisturizing factor (NMF), the optimal function of physiological and mechanical skin properties, as well as cutaneous metabolism and enzyme function.^9^, ^10^, ^11^ An adequate moisturization of the SC is also essential for the healthy appearance of the skin. Maintenance of SC hydration is dependent on factors such as relative atmospheric humidity, the capability to balance evaporative water loss, the presence of extracellular lipids and the ability of corneocytes to bind water.^12^

This water‐binding capacity is dependent on NMF, a complex mixture of low‐molecular‐weight, highly hygroscopic, water‐soluble compounds that are exclusively present in the SC.^9^ NMF mainly consist of free amino acids, pyrrolidone carboxylic acid, lactates, citrate, sugars, and urea, forming a depth profile that peaks in the upper third of the SC.^5^, ^13^ Reductions or a deficiency in NMF have been associated with various skin conditions such as xerosis cutis.^13^

To increase the diminished SC water content, moisturizers containing glycerol or urea are well established.^13^, ^14^ Glycerol enhances SC hydration, prevents epidermal water loss as well as SC thickening, and improves dry, scaly skin.^15^, ^16^, ^17^ However, urea is currently considered the gold standard in the treatment of dry skin^18^, ^19^ since it has been demonstrated to improve hydration mechanisms and skin barrier function and its safety and efficacy have extensively been documented over the last century.^18^, ^19^, ^20^, ^21^ The efficacy of urea can be further enhanced by the addition of other NMF components, and glyceryl glucoside and glycerol.^19^, ^22^, ^23^

The aim of this study was to investigate the objective and subjective hydrating efficacy of a moisturizer formulation containing these humectants compared to its emulsion base containing glycerol (referred to as the vehicle). The effects of formulations containing 10% urea on skin hydration have repeatedly been confirmed by us and others in vehicle‐controlled studies using capacitance‐based corneometry.^20^, ^23^, ^24^, ^25^, ^26^, ^27^ Several investigations applying confocal Raman microspectroscopy in vitro and in vivo have elucidated the depth profile of SC water content in detail.^7^, ^8^, ^28^, ^29^ Here, we applied the recently developed near‐infrared confocal spectroscopic imaging (KOSIM IR) method that allows a noninvasive measurement of skin hydration in vivo as a function of skin depth^6^ to detect depth‐dependent alterations in water content and related the results to Corneometer^®^ values.

## 2 MATERIALS AND METHODS

### 2.1 Test formulations

The moisturizer formulation (Eucerin^®^ Urea Repair PLUS Lotion 10%, Beiersdorf AG, Hamburg, Germany) contained 10% urea, supplementary NMF components (lactic acid, sodium lactate, amino acids), and glyceryl glucoside. The moisturizer and vehicle formulation likewise contained glycerol.

Ingredients of the formulations according to INCI:

Moisturizer containing actives: Aqua, Urea, Glycerin, Isopropyl Stearate, Dicaprylyl Ether, Glyceryl Glucoside, Sodium Lactate, Butyrospermum Parkii Butter, Polyglyceryl‐4 Diisostearate/Polyhydroxystearate/Sebacate, Tapioca Starch, Carnitine, Cetearyl Alcohol, Ceramide NP, Arginine HCL, Sodium PCA, Histidine HCl, Lactic Acid, Mannitol, Arginine, Serine, Sucrose, PCA, Citrulline, Glycogen, Alanine, Threonine, Glutamic Acid, Lysine HCl, Sodium Chloride, 1,2‐Hexanediol, Phenoxyethanol, Potassium Sorbate.

Vehicle: Aqua, Glycerin, Isopropyl Stearate, Dicaprylyl Ether, Butyrospermum Parkii Butter, Polyglyceryl‐4 Diisostearate/Polyhydroxystearate/Sebacate, Tapioca Starch, Cetearyl Alcohol, Ceramide NP, 1,2‐Hexanediol, Phenoxyethanol, Potassium Sorbate.

### 2.2 In vivo studies I and II

For both single‐center, randomized, single‐blind, vehicle‐controlled studies, the recommendations of the current version of the Declaration of Helsinki and the guidelines of the International Conference on Harmonization of Good Clinical Practice (ICH GCP) were observed as applicable to a non‐drug study. All volunteers provided written, informed consent.

#### 2.2.1 Study I: Corneometer measurements and self‐assessments of skin moisturization

This short‐term study was conducted at the Dermostica Institute Bartosz Walis, Poznań, Poland. Forty‐two healthy, female volunteers (19–64 years old, mean age 38.6 years) with self‐reported dry to very dry lower leg skin belonging to the Fitzpatrick Skin Classification Types I to III^30^ were enrolled in the study.

Five days prior to the study start, subjects were asked to refrain from using skin care products. Measurements were carried out by trained and experienced personnel after acclimatization for 30 min under standard atmospheric conditions (21.0°C ± 1.0°C and 50% ± 5% relative humidity).

Skin moisturization of the lower legs was assessed using a Corneometer^®^ CM 825 (MDD4 device, Courage and Khazaka, Cologne, Germany). Ten measurements were performed per test site according to the European Group for Efficacy Measurements on Cosmetics and Other Topical Products (EEMCO) guidelines.^31^ Also, subjects assessed their skin condition by using a self‐grading questionnaire. Volunteers were requested to observe their skin and grade skin moisturization by means of a scale ranging from 1 (extremely dry and scaly) to 10 (extremely moisturized, no signs of dryness). Under supervision of the investigator subjects then treated one of their lower legs with the moisturizer and the other one with the vehicle, applying approximately 2 g each. Corneometer^®^ measurements and self‐gradings were repeated 30 min and 24 h after application. Twenty‐four hours after application, volunteers additionally evaluated product performance using a two‐tiered rating scale: 1 = no and 2 = yes. If a volunteer skipped a question, the answer was saved as 0 = I do not know.

#### 2.2.2 Study II: KOSIM IR measurements

A total of 27 healthy, female volunteers (24–55 years old, mean age 38.1) with self‐reported normal skin, Fitzpatrick Skin Classification Types I to III were enrolled into this study conducted at the Beiersdorf Test Center, Hamburg, Germany.

During a 7‐day preconditioning period and during the entire study, volunteers were required to refrain from using skin care products on their arms. Subsequent assessments of baseline skin hydration by corneometry indicated dry skin conditions in the designated test areas for application of moisturizer or vehicle and the untreated control on inner forearms (26.2 ± 5.2, 27.1 ± 6.2, and 26.6 ± 5.0 a.u., respectively).^12^

Measurements were performed by trained and experienced personnel after acclimatization for at least 30 min under standard atmospheric conditions (21.5°C ± 1.0°C and 45% ± 5% relative humidity). Depth‐resolved water‐absorption spectra were assessed using KOSIM IR at baseline and after 14 days of twice daily application.

#### 2.2.3 KOSIM IR

KOSIM IR was developed by Beiersdorf AG in cooperation with 4DOS GmbH (Hamburg, Germany) and is a method that combines near‐infrared spectroscopy and confocal microscopy for selective, depth‐resolved determination of the mass percentage of water in different skin layers with high spatial resolution.^6^, ^19^ Starting from the skin surface this spectroscopic system scans to a designated depth of 100 μm, using a depth‐resolution of 2.5 μm. Each measurement consisted of nine water‐profiles covering an area of 4 × 4 mm. At least five measurements were performed per test site. In the near‐infrared region, KOSIM IR covers a spectral range from 860 to 2010 nm and the water absorption bands at 1450 and 1950 nm were analyzed. The KOSIM IR measures the complete NIR response covering these two absorption bands. The fit of the spectra as described by Behm et al^6^ allows to discriminate the general changes in optical properties of skin compared to the absorption amplitudes of water. Since both absorption bands yield identical results, we can validate this approach for each measurement. Since the bands at 1450 and 1950 nm have no relevant contribution from ‐CH, we can determine the absorption amplitudes specific to H_2_O by knowing the confocal resolution of the instrument. These changes in H_2_O absorption can be translated to changes into the water content in m% by inverting Lambert–Beers Law.

$$\Delta c\;\left[m\%\right]=-\frac{1}{\eta \Delta z}\ln\left(\frac{A\left(\Delta z\right)}{\left(A_{0}\right)}\right)$$

Δ*c*: *denotes the change in water concentration*
η: *is the absorption coefficent in* $\frac{1}{\mu m}$
Δ*z*: *is the change in z‐direction*
*A*: *denotes the amplitude in z as function of depth*
*A*_0_: *denotes the amplitude at the surface of skin*

**FIGURE 1:**
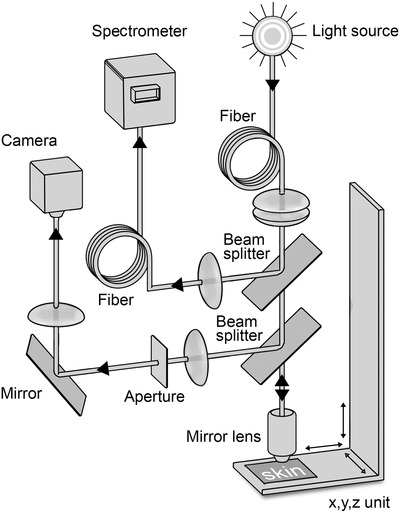
KOSIM IR System consisting of a real time imaging branch and a synchronized spectroscopic unit covering the spectral range between 810 and 2100 nm; for details refer to Behm et al^6^

**FIGURE 2:**
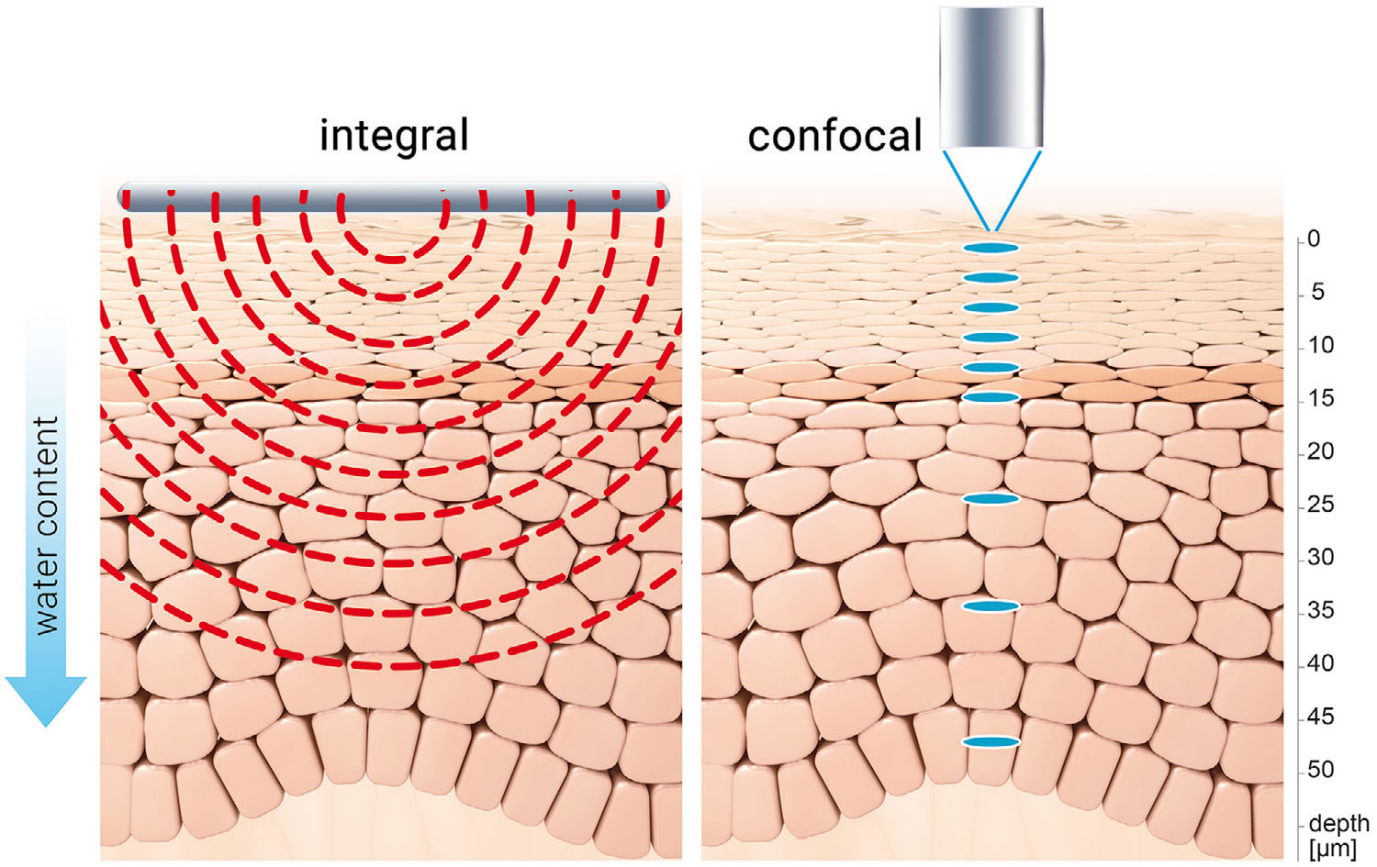
Comparison of measurement principles of Corneometer^®^ (integral) and KOSIM IR (confocal) assessment of skin hydration. Corneometer^®^ integrates skin hydration values to a depth of 45 μm while KOSIM IR provides a depth‐resolved determination of mass percentage of water with high spatial resolution to a depth of 100 μm

The measured value was defined as: mass percent water [% × 100] as function of depth [μm]. The technical system is illustrated in Figure 1 and the measurement principle compared to corneometry in Figure 2.

### 2.3 Statistical analysis

Statistical analyses were performed using Microsoft Excel for Office 365, SAS Software Package for Windows V9.4 (SAS Institute GmbH, Heidelberg, Germany) and STATISTICA 10.0 (StatSoft, Inc., Tulsa, U.S.). All statistical tests were two‐sided at significance level alpha = 0.05.

#### 2.3.1 Study I: Corneometer measurements and skin moisturization assessments

Data were tested for conformity with a normal distribution using Shapiro–Wilk's test. In the case when the conformity was absent, a logarithm transformation of the data was performed. Statistical analysis utilized pairwise Wilcoxon's signed rank test (when conformity of data with a normal distribution was absent even after transformation). Statistical analysis of the self‐grading results employed nonparametric pairwise Wilcoxon's signed‐rank test. For results of self‐assessment questionnaires, frequency of volunteer answers was calculated. For two‐tiered and three‐tiered rating scales binomial test and sign test according to Dixon‐Mood were conducted.

#### 2.3.2 Study II: KOSIM IR measurements

Data were tested for conformity with a normal distribution using Shapiro–Wilk's test. If the normality hypothesis was rejected, analysis of the Blom‐transformed ranks of the original data was done. Otherwise, the original data were analyzed. Evaluation of treatment effects was done by analysis of variance. In all statistical models, a volunteer effect for repeated measurements was considered.

The area under curve (AUC) was calculated as follows:

$$\begin{array}{ccc} AUC & = & \frac{x_{0}+x_{2.5}}{2}2.5+\frac{x_{2.5}+x_{5}}{2}2.5+\frac{x_{5.0}+x_{7.5}}{2}2.5\\ & & +\;\frac{x_{7.5}+x_{10}}{2}2.5+\frac{x_{10.0}+x_{15.0}}{2}5\\ xi & = & \;\text{mass}\;\text{percentage}\;\text{water}\;\text{at}\;\text{depth}\;i \end{array}$$

## 3 RESULTS

### 3.1 Study I

#### 3.1.1 In vivo determination of skin moisturization using corneometry

**FIGURE 3:**
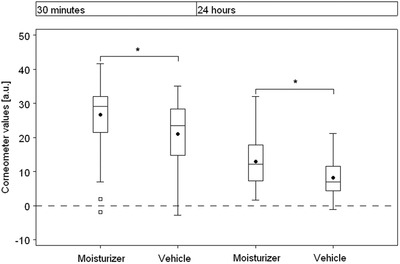
Skin moisturization of dry lower leg skin was measured by corneometry 30 min and 24 h after a single application of the moisturizer and the vehicle (n = 42). Box plots of differences to baseline. Results are depicted as mean ± SD. The dot (⚫) corresponds to the mean and the line (‐) corresponds to the median. 50% of the values are inside the box. Significant differences are marked with an asterisk (*p < 0.05).

As illustrated in Figure 3, compared to baseline values (23.66 ± 7.60 a.u.), legs treated with the moisturizer (50.32 ± 8.59 a.u.) showed a statistically significant increase in corneometry values 30 min after single application (*p* < 0.0001). At the same point in time, legs treated with the vehicle demonstrated, compared to baseline (24.20 ± 7.53 a.u.), also a significant augmentation (45.21 ± 9.57 a.u., *p* < 0.0001). Twenty‐four hours after application, corneometry values of legs treated with the moisturizer were yet significantly increased to 36.55 ± 10.45 a.u. (*p* < 0.0001). Application of the vehicle also resulted in a significant augmentation of values to 32.34 ± 9.44 a.u. (*p* < 0.0001).

Compared to the vehicle, application of the moisturizer resulted in statistically significant higher corneometry values at both points in time (30 min after application: *p* < 0.001, 24 h after treatment: *p* < 0.0001).

#### 3.1.2 Volunteers’ subjective assessment of skin hydration

Subjects rated the skin hydration of the lower leg skin treated with the moisturizer at baseline with a mean value of 3.90 ± 1.45. Thirty minutes after application of the moisturizer this value significantly (*p* < 0.0001) increased to 8.10 ± 1.78 and, 24 h after treatment, to 7.93 ± 1.69 (*p* < 0.0001).

Compared to baseline values (3.95 ± 1.62) subjects assessed the hydration of skin treated with the vehicle as significantly (*p* < 0.0001) improved after 30 min (8.14 ± 1.62) and, also after 24 h (7.40 ± 1.68). Legs treated with the moisturizer received statistically significant higher ratings with respect to skin hydration 24 h after treatment (*p* < 0.05).

**FIGURE 4:**
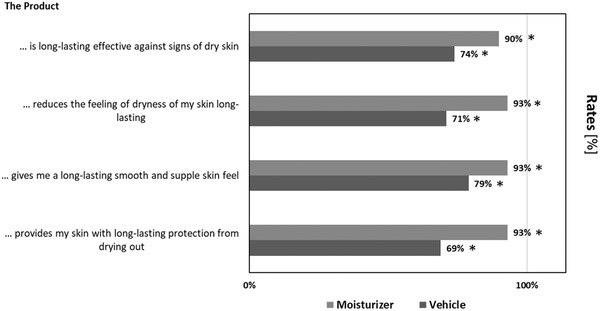
Self‐assessment of skin hydrating product properties 24 h after a single application of the moisturizer and the vehicle (n = 42). Results are depicted as confirmation rates. Significant agreement rates are marked with an asterisk (*p < 0.05).

Figure 4 illustrates the results of the self‐assessment of the skin hydrating properties of moisturizer and vehicle by the volunteers 24 h after treatment.

Additionally, according to the self‐assessment questionnaire 64% of subjects rated the moisturizer as more effective against signs of dry skin than the vehicle (22% preference), significantly favoring the moisturizer (*p* < 0.01).

### 3.2 Study II

#### 3.2.1 In vivo determination of skin moisturization using KOSIM IR

**FIGURE 5:**
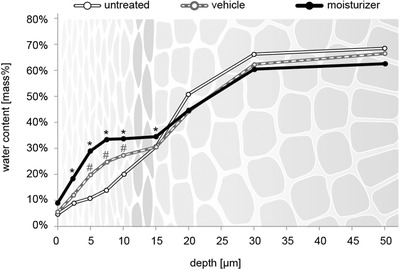
Water concentration profiles in the SC of dry forearm skin in m% versus depth determined using near‐infrared confocal spectroscopic imaging (KOSIM IR) after 14 days of regular treatment with the moisturizer (n = 27) or the vehicle (n = 26). Significant differences are marked with an asterisk (*p < 0.05 comparison to vehicle; ^#^ *p* < 0.05 comparison to untreated control).

Figure 5 depicts the hydration profile of SC and viable epidermis to a depth of 50 μm. Compared to the untreated area, the application of the vehicle resulted in a significantly higher water content in the SC at depths of 5, 7.5, and 10 μm (Table 1). The moisturizer induced a significantly higher water content in the SC at all depths compared to both the untreated area and the vehicle. Moreover, the AUC revealed a significantly higher water content after treatment with the moisturizer compared to the vehicle. AUC values of sites treated with the moisturizer or the vehicle were significantly higher in comparison to control sites (Table 1).

**TABLE 1:** Study II: Mass percentage water after 2 weeks of regular application of the moisturizer and the vehicle in forearm skin

	**Mass percentage water (%), mean values of original data ± SD**		
	**Untreated control (n = 27)**	**Vehicle (n = 26)**	**Moisturizer (n = 27)**
0 μm	5.8 ± 3.7	5.8 ± 3.8	8.9 ± 3.6 *p* (comparison to control) = 0.0089 *p* (comparison to vehicle) = 0.0079
2.5 μm	10.2 ± 5.8	12.3 ± 6.8	18.7 ± 6.5 *p* (comparison to control) = 0.0002 *p* (comparison to vehicle) = 0.0033
5 μm	11.9 ± 6.5	19.9 ± 8.8 *p* (comparison to control) = 0.0012	29.1 ± 7.1 *p* (comparison to control) = 0.0000 *p* (comparison to vehicle) = 0.0001
7.5 μm	14.4 ± 6.1	24.3 ± 7.7 *p* (comparison to control) = 0.0001	33.1 ± 7.3 *p* (comparison to control) = 0.0000 *p* (comparison to vehicle) = 0.0000
10 μm	20.0 ± 5.9	27.3 ± 6.1 *p* (comparison to control) = 0.0002	33.4 ± 6.7 *p* (comparison to control) = 0.0000 *p* (comparison to vehicle) = 0.0037
15 μm	30.8 ± 6.5	30.1 ± 4.2	33.6 ± 6.8 *p* (comparison to vehicle) = 0.0320
AUC (% * μm)	250.4 ± 73.4	326.1 ± 71.5 *p* (comparison to control) = 0.0017	422.3 ± 67.2 *p* (comparison to control) = 0.0000 *p* (comparison to vehicle) = 0.0000

## 4 DISCUSSION

In the studies reported here, we assessed the hydrating capacity of a moisturizer containing 10% urea, supplementary NMF components, glyceryl glucoside, and glycerol in comparison to its vehicle containing glycerol in dry skin by applying two different methods. Corneometry is a well‐established capacitance method that provides a single dielectric value (arbitrary units) of skin hydration per measurement, integrating skin hydration values to a depth of 45 μm.^31^, ^32^

The Corneometer^®^ assessments revealed statistically significant higher values after application of the moisturizer, compared to the vehicle, 30 min and 24 h after treatment, indicating an instant and sustainable effect on skin hydration. These findings were reflected by the volunteers` ratings of the skin hydrating properties of the moisturizer, resulting in significant confirmation rates. Accordingly, the moisturizer was significantly more frequently rated as effective against signs of dry skin than the vehicle. A high level of acceptance is critical for treatment adherence that depends on patient preference.^33^ Moreover, these findings corroborate an earlier vehicle‐controlled study of the moisturizer in subjects with very dry skin that evidenced the effect of the applied humectants on skin hydration as paralleled by improvements in clinical severity grading.^23^ Accordingly, NMF components are regarded crucial for the maintenance of adequate skin hydration in the treatment of xerosis and other skin conditions.^13^, ^14^, ^19^

To investigate influences of the moisturizer and its vehicle on SC hydration dynamics in more detail, we furthermore assessed the intracorneal water gradient by KOSIM IR. This novel method determines the depth‐dependent hydration profiles of human skin in vivo and can be employed in areas where the dielectric based readings of the classical Corneometer^®^ reach their technical limits.^6^

At baseline and after 2‐week application, water concentration depth profiles in the SC were revealed at high spatial resolution. Treatment with the moisturizer resulted in significantly higher hydration levels, compared to the vehicle, throughout SC to a depth of 15 μm. Below the stratum lucidum/stratum granulosum interface at approximately 20 μm, maximum hydration levels were observed in all investigated skin areas (Figure 5).

These results are in line with water concentration profiles of normal and hydrated skin obtained by confocal Raman microspectroscopy that displayed a gradual increase in hydration levels as a function of SC depth in vivo^7^, ^8^, ^28^ and significant larger water inclusions in skin pretreated with a urea solution in vitro.^29^ In dry skin, SC water concentration profiles are paralleled by characteristic differences in protein and free amino acid depth profiles that display decreased NMF levels at the same depth.^34^ Also, individuals who are carriers of FLG‐null mutations showed, compared to FLG carriers, significantly reduced levels of NMF at all SC depths.^35^

A recent KOSIM IR study analyzed the cutaneous hydration dynamics before and after treatment with glycerol and demonstrated augmented water concentration gradients in the SC proportional to increasing glycerol concentrations.^6^ It was also revealed that results obtained from corneometry and KOSIM IR correlated well in normal skin. In our investigation, a similar relationship was demonstrated, for the first time, also in dry skin. While the significant and profound hydrating effects of the vehicle may be attributed to its content in glycerol, an additive effect on skin hydration and SC water concentration gradients was demonstrated for the combination of 10% urea with supplementary NMF components and glyceryl glucoside. In both skin conditions corneometry delivers quantitative data on SC bulk water content whereas KOSIM IR provides qualitative information in high resolution about the water concentration gradient, rendering both methods complementary.

## 5 CONCLUSION

Taken together, our results demonstrate that the moisturizer containing 10% urea, supplementary NMF components, glyceryl glucoside, and glycerol improved SC hydration in total and, moreover, as a function of depth. The moisturizer provided a superior hydrating effect compared to the vehicle containing glycerol in vivo in dry skin. The volunteers perceived the moisturizer as superior in the treatment of dry skin compared to the vehicle. This correlation between objective and subjective data suggests a positive effect also on patients` treatment adherence. Application of KOSIM IR in combination with provides a novel, holistic approach to the measurement of skin hydration.

## CONFLICT OF INTEREST

Julia Gallinger, Andreas Kuhn, Sonja Wessel, Peter Behm, Silke Heinecke, Alexander Filbry, Linus Hillemann, and Frank Rippke are employees of Beiersdorf AG.
